# An update and reassessment of fern and lycophyte diversity data in the Japanese Archipelago

**DOI:** 10.1007/s10265-019-01137-3

**Published:** 2019-09-16

**Authors:** Atsushi Ebihara, Joel H. Nitta

**Affiliations:** 1 Department of Botany, National Museum of Nature and Science, 4-1-1 Amakubo, Tsukuba, Ibaraki 305-0005 Japan; 2 Department of Botany, National Museum of Natural History, Smithsonian Institute, Washington, DC 20013 USA

**Keywords:** Interactive keys, Occurrence data, Phylogenetic diversity, Species richness

## Abstract

The fern and lycophyte flora of Japan comprising 721 native taxa (including subspecies and varieties) plus 371 interspecific hybrids was reassessed using a nearly comprehensively sampled distribution map at 10 km resolution vouchered by 216,687 specimens, up-to-date cytotaxonomic information covering 74% of the taxa, and an *rbcL* sequence dataset covering 97.9% of the taxa. Spatial distribution of species richness and phylogenetic diversity was visualized. Apomixis was observed in 11.0% of the native taxa whose reproductive modes are known. The number of sexually reproducing polyploid taxa (*n* = 199) is less than sexual diploids (*n* = 241), and 30 of them are evidently allopolyploid, in contrast with the low number of possible autopolyploids (*n* = 4). Apomictic taxa were found to have smaller latitudinal ranges than sexual taxa or taxa with multiple reproductive modes. A morphological character dataset in Lucid format is provided for taxonomic identification of the native taxa.

## Introduction

Ferns and lycophytes, also traditionally known as ‘pteridophytes’, have received much attention as biological research material. Although there are only estimated to be 11,916 extant species of ferns and lycophytes (Pteridophyte Phylogeny Group 2016)—much less than the number of seed plant species (approximately one twelfth)—the number of studies on ferns and lycophytes, especially in the field of systematics and evolution, is relatively large. Thus, ferns and lycophytes are relatively information-rich organisms. However, examples of analyses of fern diversity patterns at the regional scale are scarce (e.g., Bogonovich et al. 2014; Mountier et al. 2018). Over the past century, the diversity of ferns and lycophytes in Japan was quite thoroughly studied, and is probably the third best studied area after Europe and North America. Guo et al. (2003) combined trait and occurrence data to analyze determinants of species diversity, abundance, and distribution of the pteridophyte flora of Japan. The sole serious shortcoming of the analyses of Guo et al. (2003) was the lack of phylogenetic information at that time, but DNA sequence data for nearly all ferns and lycophytes of Japan (at least for the chloroplast) have been produced by subsequent studies (Ebihara 2011; Ebihara et al. 2010). In addition, it should be noted that the occurrence data based on Kurata and Nakaike (1979, 1981, 1983, 1985, 1987, 1990, 1994, 1997) used by Guo et al. (2003) did not cover all known species at the time, and more have been revised or added since. Ebihara (2016, 2017) provided updated distribution maps for all the native taxa of ferns and lycophytes in Japan reflecting numerous new occurrences and recircumscription of taxa based on the results of recent studies, but did not make the data available online. In the current study, we make available the most recent diversity datasets on ferns and lycophytes in Japan, review recent progress and outstanding questions in documenting this flora, and reassess the results of Guo et al. (2003).

## Flora

Study on the fern and lycophyte flora of the Japanese Archipelago began with 44 species enumerated in Thunberg (1786)’s “Flora Japonica”. Thereafter the number of recognized native taxa has been gradually increasing: 460 species including subspecies and varieties (Makino and Nemoto 1925), ca. 400 species excluding those in Ogasawara (Bonin) and Ryukyu Islands (Tagawa 1959), and 630 species (Iwatsuki et al. 1995). The latest checklist of native taxa of Japan, which we use in this study, is the ‘FernGreenList ver. 1.01’ (in Japanese; Ebihara et al. 2017a). It comprises 687 species (721 taxa including subspecies and varieties; hereafter called ‘taxa’) belonging to 37 of the 51 families accepted in PPGI (Pteridophyte Phylogeny Group 2016) (ESM 1). As almost no uninvestigated area remains in the archipelago, most of the new additions to the flora after the 1990s have been newly segregated species from previously known ones based on evidence from ploidy levels, enzyme analyses and/or DNA sequences (e.g., Ebihara et al. 2019b; Fujiwara et al. 2018; Hori et al. 2015, 2018a, 2018d; Lin et al. 1994, 1996; Masuyama and Watano 2010; Murakami et al. 1999; Ohta and Takamiya 1999; Serizawa 2015; Takamiya et al. 1997) along with only a small number of new records (Ebihara et al. 2014, 2019a). The number of taxa in Japan is similar to that of Taiwan (Knapp 2014; Taiwan Pteridophyte Group 2019). The most recent information as of writing identified 125 endemic taxa (17.3% of the native taxa) to Japan (Ebihara 2018).

## Historical biogeography

The area with the strongest floristic affinity to the fern and lycophyte flora of Japan is continental China, with which Japan shares 487 taxa (67.5%), corresponding to nearly two-thirds of the native taxa in Japan (Ebihara 2016, 2017). Together, Japan and continental China belong to the Sino–Japanese floristic region (Takhtajan 1986) proposed chiefly based on seed plant distribution patterns. Despite this affinity, few studies have been carried out including the ferns and lycophytes of continental China and Japan in the context of historical biogeography. Isagi (2012) analyzed all the extant individuals of *Diplazium pinfaense* Ching in four locations in Kyushu using SSR markers, and suggested that the four populations each originated from a single spore, probably by long distance dispersal from the continent. A certain number of species with similar distribution pattern—widely distributed in China but only one or a few localities in Central/Western Japan—might have dispersed into the Japanese Archipelago by long-distance spore dispersal from the continent. Although the number of common elements is not nearly as many as the Sino–Japanese ones, the eastern Asian–North American disjunct distribution pattern of vascular plants has received attention since the nineteeth century (e.g., Gray 1859), and a number of examples of disjunct distributions are found in ferns and lycophytes between these areas (Kato 1993; Kato and Iwatsuki 1983). Nevertheless, historical biogeographic scenarios based on appropriate sampling and phylogenetic methods have been suggested only for a few species groups (reviewed by Xiang et al. 2015). In the *Adiantum pedatum* group, two intercontinental migration events via land bridges were inferred (Asia to North America in the Pliocene–Pleistocene, and North America to Asia in the Pleistocene; Lu et al. 2011). Kuo et al. (2016) estimated the divergence time of *Deparia acrostichoides* (Sw.) M. Kato endemic to eastern North America from its closest relative in Asia (the *D. pycnosora* group), suggesting that it originated by vicariance in the Miocene.

## Interspecific hybrids

The great number of interspecific natural hybrid taxa (371 combinations listed in Ebihara et al. 2017a) known in addition to the 721 native, non-hybrid taxa is an outstanding characteristic of the Japanese pteridophyte flora. This is much more than the number of known hybrid combinations in China (10 were formally accepted in Flora of China (Wu et al. 2013), and 20 or more combinations were suggested in the notes), or Taiwan (31 listed in Taiwan Pteridophyte Group 2019). Such high diversity is most likely a result of efforts seeking new hybrid combinations by Satoru Kurata (1922–1978) and his pupils, but might be, at least in part, a geographical effect of the long north–south axis of the Japanese archipelago, which may enable sympatric occurrence of species with different habitat preferences concomitant with climate fluctuation. It is highly likely that most of the interspecific hybrid individuals in a large part of the world are yet unrecognized, including cases of misidentification as non-hybrid taxa.

It should be noted that only a few combinations of hybridity in Japan have been verified by artificial crossing, enzyme patterns, or nuclear DNA markers; the others are putative hybrids based on morphology. An expedient method to identify F_1_ hybrids is to observe the size and shape of spores; ill-formed or aborted spores are often due to failure of chromosomes from divergent species to properly pair at meiosis (e.g., Wagner 1954; Wagner and Chen 1965). However, this is not always reliable in the case of homoploid hybrids, which often have normal-shaped, regular spores, e.g., *Botrychium* × *silvicola* (Sahashi) Ebihara (Ebihara et al. 2015; Sahashi 1983) and other putative homoploid hybrids of *Botrychium* species in Japan (N. Sahashi, personal communication).

Studies on hybrids in Japan including more thorough analysis beyond observations of spores and morphology provide insights into their evolutionary dynamics. An incomplete establishment of reproductive isolation was observed between *Osmunda lancea* Thunb., a rheophytic species endemic to Japan, and its closest relative *O. japonica* Thunb. (Yatabe et al. 2009, 2011). Their ‘hybrid’ *O.* × *intermedia* (Honda) Sugim. actually consists of hybrid swarms including not only F_1_ but also F_2_ or later-generation hybrids. In case of *Thelypteris* (*Stegnogramma*) *pozoi* (Lag.) C. V. Morton subsp. *mollissima* (Fisch. ex Kunze) C. V. Morton, two morphologically distinct diploid forms have been classified into a single taxon on the basis of the complete fertility of their artificial hybrids (Yatabe et al. 2002). A recent plastid phylogeny of *Stegnogramma* s.l. (Kuo et al. 2019) highlighted the non-monophyletic relationships and extraordinary phylogenetic distances within *T. pozoi* subsp. *mollissima*, and further careful study is awaited. Fern interspecific hybrids have long been believed to occur in places where sporophytes of their two parent species are sympatric, and this idea was an important grounds for identification of putative parentages. However, our studies demonstrated a number of examples of sterile interspecific hybrids unaccompanied by their parental species. One of the extreme cases is found in the dennstaedtioid fern *Monachosorum* × *arakii* Tagawa, which was originally considered as an endemic species to Japan. Ebihara et al. (2016) concluded that it is a sterile hybrid between *Monachosorum henryi* Christ and *M. nipponica* Makino. The former is currently distributed in Taiwan, continental China, and Himalaya, but not Japan. Although *M.* × *arakii* is a somewhat special case with strong vegetative reproduction ability—it can asexually reproduce from both rhizomes and frond-borne bulbils—making its relict distribution possible, we should keep in mind that current species distributions may not restrict the possible combinations of putative parents when constructing hypotheses of hybrid origins. Apart from relict distribution, there is also strong evidence for the contribution of independent gametophytes to formation of hybrid sporophytes in *Vandenboschia* in Japan (Ebihara et al. 2009a).

## Occurrence data and alpha diversity

The eight volumes of “Illustrations of Pteridophytes of Japan” (Kurata and Nakaike, 1979, 1981, 1983, 1985, 1987, 1990, 1994, 1997) were a remarkable achievement of citizen-scientists to document species distributions at high resolution throughout an entire country. In addition to a brief description of each species, these books featured ca. 10 km × 10 km grid-cell distribution maps (hereafter, “10 km grid-cell maps”) based on vouchers of ca. 180,000 specimens collected by the members of the Nippon Fernist Club (NFC). Surveys of the NFC have continued to the present, and a large part of the specimens presently deposited in the herbarium of the National Museum of Nature and Science (TNS) were recently re-identified accommodating up-to-date species circumscriptions and new collections by club members, resulting in an updated set of 10 km grid-cell maps including ca. 35,000 new grid-cell records (Ebihara 2016, 2017). Occurrences based on specimens are usually regarded as presence-only data, but the distribution maps of Ebihara (2016, 2017) can be virtually regarded as presence/absence data due to the extensive collection effort of NFC members. For the convenience in accessing and reusing the data, we make the data used to construct the distribution maps in Ebihara (2016, 2017) available here as a spreadsheet of species occurrences (presence-only) per grid-cell (216,687 rows; ESM 2). Our policy for the dataset is to only include occurrences based on specimens or specimen-images examined by ourselves (the only exception is *Isoëtes* occurrences, which are cited from a specimens list provided by Takamiya et al. (1997). At least one species is recorded in 4342 out of 4852 grid-cells total (89.5%), and the most abundant species is *Pteridium aquilinum* (L.) Kuhn subsp. *japonicum* (Nakai) Á. Löve et D. Löve (Dennstaedtiaceae), which was recorded in 2777 grid-cells (57.2%).

Although not included in the present dataset, a larger number of specimen records without images are available on-line via Science Museum Net, the local data portal for museum collections in Japan (http://science-net.kahaku.go.jp/), and the Global Biodiversity Information Facility (GBIF) data portal (https://www.gbif.org/). As of May 23, 2019, Japan is the country with the largest number of fern and lycophyte specimens in GBIF (531,311 occurrences).

**Fig. 1:**
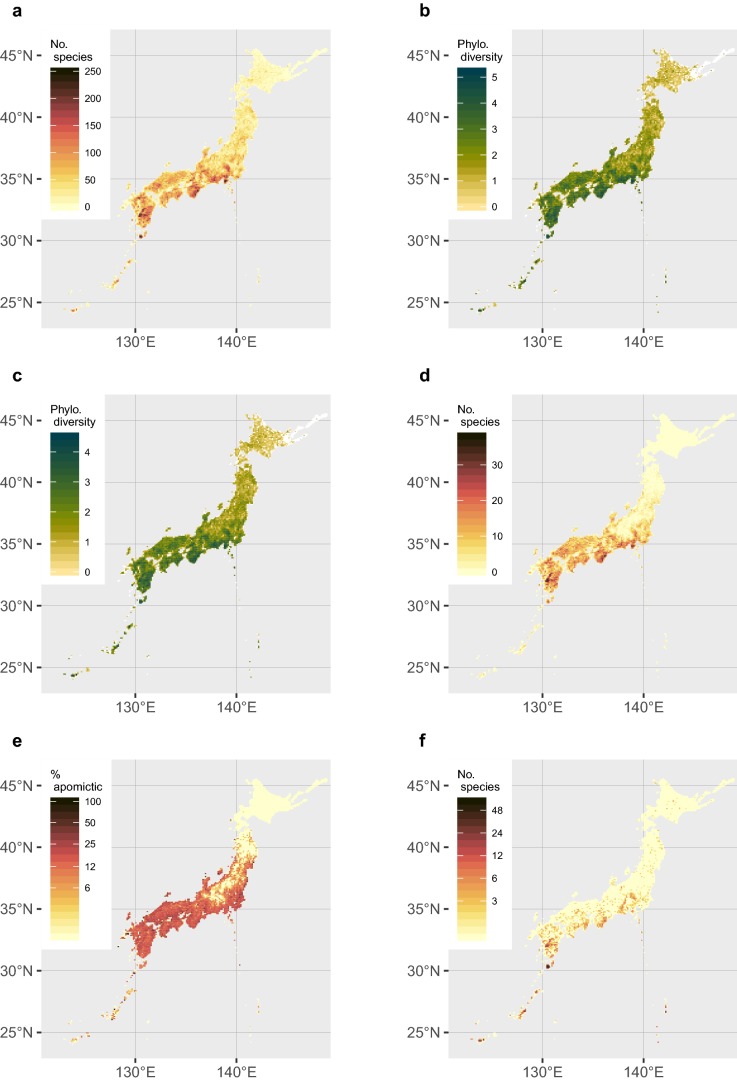
Maps of fern and lycophyte diversity in Japan on a 10 km × 10 km grid. Taxa used to calculate diversity metrics include native species, subspecies, and varieties; **a**, **f** also include hybrids, while **b**–**e** do not. **a** Richness of native fern and lycophyte taxa. **b** Phylogenetic diversity of fern and lycophyte taxa. **c** Phylogenetic diversity of fern taxa. **d** Richness of apomictic fern taxa. **e** Proportion of apomictic taxa out of all taxa. **f** Richness of threatened fern and lycophyte taxa included in the national red list of Japan. Note that the color gradients for **e** and **f** are on a log-scale. For **b** and **c**, units are total branch-lengths of the phylogenetic tree (number of expected changes per nucleotide); white indicates PD not defined (not enough species present to calculate PD)

Species richness per 10 km grid-cell excluding nothotaxa is shown in Fig. 1a. The grid-cells with the highest (216 taxa, grid code: 453034) and the second highest (214 taxa, grid code: 453044) richness are found in the mountainous area of Yakushima Island, located south of Kyushu. The third highest grid-cell (204 taxa, grid code 503547) is located in the area covering Nachi-no-taki Falls in southern part of Wakayama Prefecture, southern Honshu.

Species richness is only one dimension of biodiversity; phylogenetic diversity is also important to consider for understanding biogeographic patterns and setting conservation priorities (Faith 1992). Datasets of the plastid *rbcL* gene and *psbA*-*trnH* intergenic spacer for ferns and lycophytes in Japan were established by Ebihara et al. (2010). We chose to use the former for phylogenetic diversity analysis because of its more uniform rate of evolution compared to the *psbA*-*trnH* intergenic spacer, which has highly variable rates of evolution in different fern lineages due to shifts into and out of the inverted repeat (Li et al. 2016). We updated the *rbcL* sequence dataset of Ebihara et al. (2010) by making several additions and corrections, which resulted in coverage of 97.9% of the native taxa (ESM 1; newly generated sequences deposited in GenBank). We aligned the sequences using MAFFT (Katoh et al. 2005), and inferred phylogenetic trees using MrBayes 3.2.6 (Ronquist et al. 2012) on the CIPRES Science Gateway (Miller et al. 2010) with 2 runs of 4 chains each for 10,000,000 generations and families according to PPG I (Pteridophyte Phylogeny Group 2016) constrained to be monophyletic. We discarded the first 25% of trees as burnin and confirmed convergence using diagnostic plots produced with the RWTY package (Warren et al. 2017) in R 3.6.0 (R Core Team 2019). We then used the majority consensus tree to calculate Phylogenetic Diversity (PD; Faith 1992; Faith and Baker 2006) for each 10 km grid-cell by trimming the tree to only taxa present in the cell and summing the branchlengths (excluding the root). We analyzed PD on a dataset including all ferns and lycophyte taxa (Fig. 1b) and a dataset including ferns only (Fig. 1c), since the deep divergence between ferns and lycophytes may bias measures of PD when lycophytes are included.

The highest PD value was obtained in a grid-cell in Wakayama Prefecture (grid code: 503547), the second highest in the northern part of the Okinawa Island (grid code: 402801) and third highest in Iriomote Island (grid code: 362346), the latter two located in the southwestern-most part of the archipelago. The grid-cell with maximum species richness (grid code 453034, in Yakushima) was ranked 10th in the PD analysis. The disparity of the results might suggest the difference in their constituent species—species richness in Yakushima Island is a result of the presence of some species-rich genera (e.g., 19 taxa of *Dryopteris*, 16 taxa of *Diplazium*, 14 taxa of *Athyrium*).

**Fig. 2:**
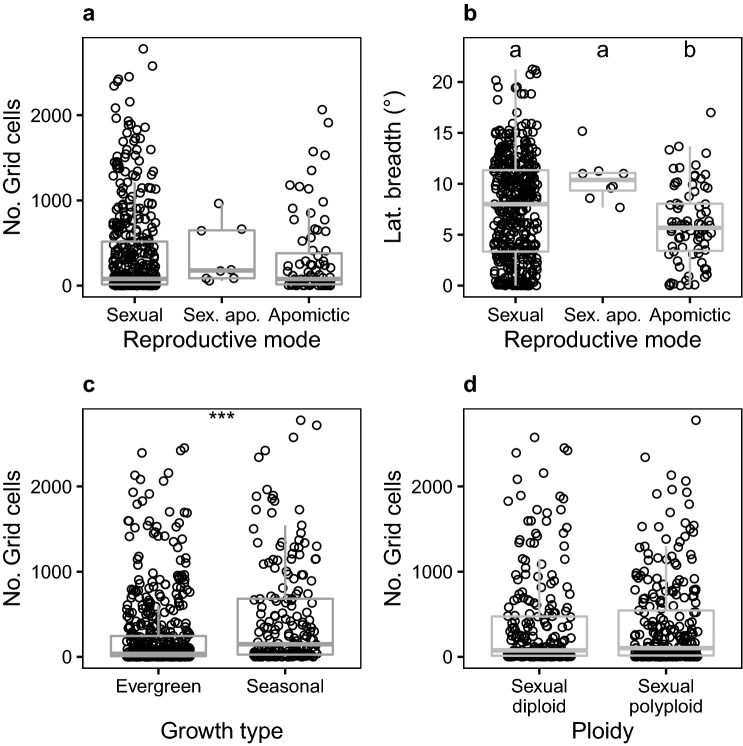
Comparisons of range size by reproductive mode, growth type, and ploidy level for the native ferns and lycophytes of Japan, excluding hybrids. Each dot represents a taxon (species, subspecies, or variety). Placement of dots randomly jittered along the x-axis to reduce overlap. **a** Distribution area (number of 10 km grid-cells) by reproductive mode [apomictic, apomictic + sexual (i.e., capable of either reproductive mode), or sexual]. **b** Latitudinal breadth (maximum latitude minus minimum latitude) by reproductive mode. **c** Distribution area by growth type (evergreen vs. seasonally green). **d** Distribution area by ploidy level (sexual taxa only; diploid vs. polyploid). For comparisons involving more than two groups (**a**, **b**), letters above groups indicate significant differences at *P* < 0.5 by Tukey’s HSD. For comparisons between two groups (**c**, **d**), asterisks indicate significant differences by Student’s *t*-test (***, *P* < 0.001). Box-and-whiskers (grey) indicate median values (bold lines); lower and upper hinges correspond to the first and third quartiles, and whiskers extend to 1.5 × the interquartile range

Guo et al. (2003) suggested that species with multiple reproductive modes are more widely distributed than species with a single reproductive mode. However, our data did not support this trend [mean numbers of grid-cells, sex. + apo. = 356.6 ± 347.3 (*n* = 8), sex. = 367.0 ± 546.8 (*n* = 413), apo. = 314.6 ± 474.7 (*n* = 79), analysis of variance, *P* = 0.73; Fig. 2a]. The result of Guo et al. (2003) seems to be strongly affected by their species circumscription, and presumably some “species with multiple reproductive modes” in Guo et al. (2003) are separated into multiple taxa each with a single reproductive mode in the present treatment. Even though they are not significantly different in the number of grid-cells they occupy, apomictic taxa show significantly narrower latitudinal ranges than sexual ones [mean latitudinal range in degrees: sex. = 7.62 ± 5.02 (*n* = 413) and apo. = 5.93 ± 3.77 (*n* = 79), *P* = 0.012, Tukey’s HSD; Fig. 2b]. This coincides with the result of Tanaka et al. (2014), who found smaller range sizes of apomicitic taxa when measured by both latitude and elevation. We also observed that the number of apomictic species tends to increase towards the south (Fig. 1d, e), in agreement with Tanaka et al. (2014), who attributed this pattern to the poor cold-hardiness of gametophytes of apomictic species due to their larger cell sizes.

Our updated occurrence data support the trend that species with seasonal-green phenology have wider distribution than evergreen species as suggested by Guo et al. (2003) [mean number of grid-cells, evergreen = 242.9 ± 443.2 (*n* = 554), seasonal-green = 458.9 ± 616.0 (*n* = 189), *P* < 0.001, *t* test; Fig. 2c]. However, we believe this is probably an artifact caused by the geography of Japan, which has larger land areas at higher latitudes (e.g., Hokkaido), since seasonal species tend to occur at higher latitudes and evergreen species at lower ones.

The trend of narrower distribution of polyploid taxa (Guo et al. 2003) was not supported by present data excluding apomictic species [mean number of grid-cells, sexual diploid = 355.9 ± 559.9 (*n* = 241), sexual polyploid = 396.1 ± 548.0 (*n* = 199), *P* = 0.752, *t*-test; Fig. 2d]. It is likely that the trend reported by Guo et al. (2003) was due to the narrower distribution of apomictic species, which are dominated by triploids.

## Cytotaxonomic data

Even today, chromosome numbers of ferns and lycophytes which are important clues for knowing ploidy level and reproductive mode, and were previously used to infer phylogenetic affinities before the introduction of DNA sequencing. Researchers in Japan, stimulated by Irene Manton’s leading work “Problems of Cytology and Evolution in the Pteridophyta” (Manton 1950), actively made chromosome counts of ferns and lycophytes using the squash technique in the second half of the twentieth century. More than 150 papers on chromosome numbers of Japanese ferns and lycophytes have been published, and all the counts on Japanese material before 1996 were indexed by Takamiya (1996). Additions have been continuously made, and Nakato and Ebihara (2016) summarized that the information drawn from Japanese individuals is available for 74% of the native taxa (61%, if nothotaxa are counted). This is a much higher level of coverage compared with other areas of East Asia [e.g., ca. 27% in China, Cheng and Zhang (2010)], and the second highest coverage in Asia following India [ca. 89%, Bir and Verma (2010)].

Further cytotaxonomic study is sorely needed for ascertaining some early records that lack voucher specimens, and for detecting infraspecific polyploidy and/or multiple reproductive modes overlooked by previous superficial sampling, as well as unsampled taxa. Flow cytometry has recently been used for inferring ploidy levels of large number of samples belonging to closely related species groups (e.g., Ebihara et al. 2005; Fujiwara et al. 2018; Hori et al. 2015; Nitta et al. 2011), but the method always requires at least one control sample whose ploidy level was confirmed by chromosome counts. Flow cytometry analyses using not only fresh leaves but also spores from recently collected herbarium specimens might accelerate data accumulation (Kuo and Huang 2017).

## Threatened species

The latest national red list of Japan (Ministry of the Environment, Japan 2019) included more than one-third of the native fern and lycophyte taxa (255 in total, 7 EX, 2 EW, 82 CR, 59 EN, 67 VU, 37 NT and 1 DD; see Fig. 1f for the distribution of richness). The largest threat for ferns is herbivory by sika deer (*Cervus nippon*) throughout the archipelago, which has clearly increased since the 1980s (Takatsuki 2009). In particular, species growing on the forest floor (e.g., *Athyrium*, Terada and Takamiya 2006) undergo severe damage, and several narrowly distributed species in Kyushu and Yakushima Island have become almost extinct or are only found inside deer fences (A. Ebihara, personal observation). Although the national law for endangered species has recently expanded the number of fern and lycophyte species which are prohibited for collection and sale (28 species as of May 2019), this is not effective for preventing herbivory by deer. There are still only a few reports on conservation of ferns and lycophytes in Japan: ubiquitous genotyping of *Athyrium viridescentipes* Sa. Kurata (Izuno et al. 2012), and clarification on the origin and propagation from spores of *Dryopteris shibipedis* Sa. Kurata (Ebihara et al. 2012), which was extinct in the wild. Despite that 73 of the 255 taxa in the latest national red list are endemic species to Japan, they are neither assessed nor listed in the IUCN Red List as globally threatened species as of May 2019 (IUCN 2019).

## Introduced species

Compared with flowering plants, the number of naturalized species of ferns and lycophytes is quite small in Japan, and they include almost no invasive species. *Selaginella uncinata* (Desv. ex Poir.) Spring (Selaginellaceae) and *Pityrogramma calomelanos* (L.) Link (Pteridaceae) are two of the scarce examples of relatively widespread introduced species in the country (Ebihara 2016). *Dryopteris carthusiana* (Vill.) H. P. Fuchs (Nakaike 2003a), *D. intermedia* (Muhl. ex Willd.) A. Gray (Nakaike 2003b) (Dryopteridaceae) and *Dennstaedtia punctilobula* (Michx.) T. Moore (Dennstaedtiaceae) (Ueno et al. 2019) were probably introduced with sprayed seeds on banks, and only collected in one or a few localities each. *Azolla cristata* Kaulf. (Salviniaceae) is the only invasive fern species controlled by national law (Invasive Alien Species Act), but is often misidentified due to morphologically similarity to an endangered and endemic species, *A. japonica* (Franch. et Sav.) Franch. et Sav. ex Nakai. Another similar species, *Azolla filiculoides* Lam., is still a matter of debate as to its natural distribution in Japan (Ebihara 2016; Suzuki et al. 2005). *Selaginella moellendolffii* Hieron. (Selaginellaceae), *Psilotum nudum* (L.) P. Beauv. (Psilotaceae), *Adiantum capillis*-*veneris* L., *Pteris vittata* L. (Pteridaceae) and *Thelypteris dentata* (Forssk.) E. P. St. John (Thelypteridaceae) are naturally distributed in the subtropical areas of the country, but are frequently found as escaped plants from greenhouses. Murakami et al. (2007) demonstrated a range expansion of *Thelypteris dentata* facilitated by urbanization in Kinki District, Japan. The natural habitat of *Cyrtomium falcatum* (L. f.) C. Presl subsp. *falcatum* is restricted to coastal areas (Ebihara et al. 2017b, Matsumoto 2003), and scattered occurrences in urban environments (Ebihara 2017) in inland areas are probably introductions.

## Reticulate evolution

Species complexes or aggregates exhibiting wide ranges of continuous morphological variation are quite commonly known in ferns and lycophytes (Haufler 1996), and this trend seems to be true for the flora of Japan as well. In case that species boundaries have become ambiguous due only to sterile F_1_ hybrids, it is usually possible to find distinct morphological gaps between progenitor species by detecting and accounting for hybrids, which often have irregular-shaped spores and low germination rates (e.g., Ohta and Takamiya 1999 in the *Diplazium mettenianum* (Miq.) C. Chr. complex). When interspecific hybrids have overcome sterility by chromosome doubling, the situation becomes more complicated (reviewed in Sigel 2016), and the biological units composing the complex are not easily clarifiable. Allopolyploids of hybrid origin often re-hybridize with their parental species—such “reticulate evolution” became well-known after the study on the Appalachian *Asplenium* complex (Wagner 1954). Similar approaches combining chromosome and morphological observations were rarely applied to Japanese species until more recently.

**Fig. 3:**
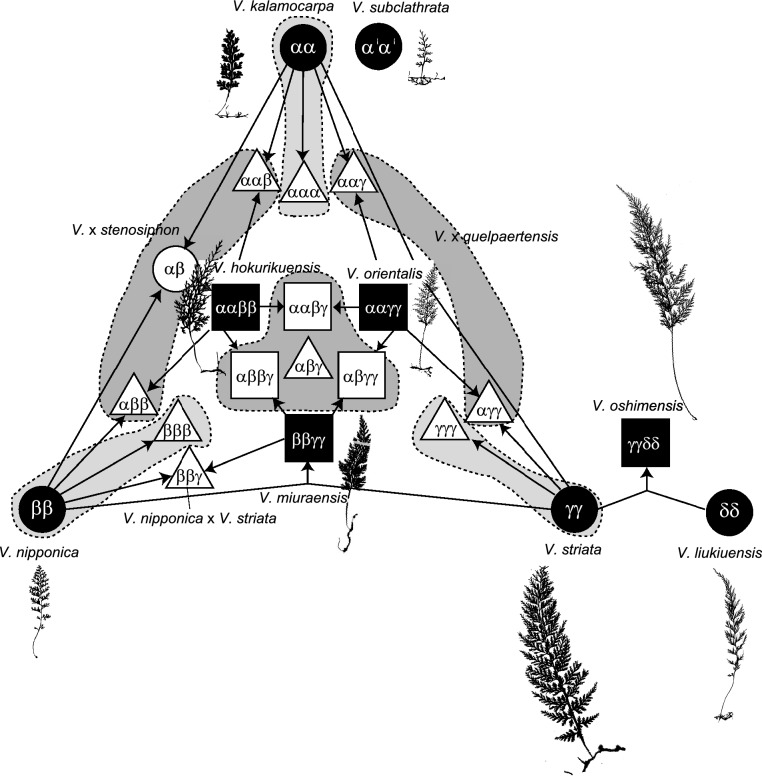
Diagram of relationships in the *Vandenoboschia radicans* complex (Hymenophyllaceae) in Japan and adjacent areas (modified from Ebihara et al. 2009b). Frond silhouettes are shown for sexual diploid and allotetraploid taxa. α, β, γ and δ each indicate a genome derived from different diploid progenitors. The shapes of symbols for genomic constitution indicate ploidy levels (circle: diploid, triangle: triploid, square: tertaploid), and the background colors indicate fertility (black: fertile, white: sterile)

Starting in the 1990s, allozyme analyses were frequently applied to fern complexes in Japan, and plastid sequences were often used as indicators of maternal lineage (e.g. Darnaedi et al. 1990 in *Dryopteris yakusilvicola* Sa. Kurata and its related species, Lin et al. 1996 in *Odontosoria*, Shinohara et al. 2010 in *Lepisorus*, Takamiya and Ohta 2001 in *Diplazium*). In the 2000s, single or low copy nuclear DNA regions became used as biparently inherited markers (e.g. Ishikawa et al. 2002), and sequence polymorphisms were identified by cloning or the SSCP (Single Strand Conformation Polymorphism) method [initiated by Ebihara et al. 2005 in the *Vandenboschia radicans* (Sw.) Copel. complex (Fig. 3), thereafter applied to various fern groups including *Ceratopteris* (Adjie et al. 2007), the *Pteris cretica* L. complex (Jaruwattanaphan et al. 2013), the *Davallia repens* (L. f.) Kuhn complex (Chen et al. 2014), and the *Asplenium normale* D. Don complex (Fujiwara et al. 2017)]. Although next-generation sequencing is widely used in phylogenetic and population genetic studies on various organisms in recent years, it is still not widely applied to analyses on species complexes of ferns and lycophytes mainly due to technical difficulties in removing chimeric contigs and phasing alleles of polyploids. More recently, Rothfels et al. (2017), Dauphin et al. (2018), and Kao et al. (2019) succeeded in phylogenetic analysis using the PacBio sequencing platform in polyploid complexes of cystoptreroid (Cytopteridaceae), moonwort (Ophioglossaceae) and notholaenid (Pteridaceae) ferns, respectively. This method is highly expected to apply to a wider range of fern species complexes.

## Species complexes involved with apomixis

Another layer of complexity is added to fern and lycophyte species complexes when apomictic reproduction (reviewed in Grusz 2016) becomes involved. Apomixis (or apogamy) occurs at a slightly higher rate in the native taxa of Japan (13% of the species whose reproductive modes are known in Takamiya 1996; 11.0% in present study) than the average rate in the world (ca. 10%, Grusz 2016). Apomixis plays a role not only in overcoming sterility of interspecific hybrids [e.g., *Athyrium christensenianum* (Koidz.) Seriz. (Park and Kato 2003)] but also enables secondary hybridization by fertilization between a sperm produced by the ‘hybrid’ and an egg of sexually reproducing races, or in the reverse direction on rare occasions (Hori et al. 2018d). This process can generate countless apomictic races with various genomic combinations which are morphologically continuous, a situation that often causes controversies about the circumscription of ‘species’ (e.g., whether to recognize a single polymorphic species versus numerous morphologically ill-defined species).

The genetic relationships of one of the apomictic complexes best known for its troublesome taxonomy, the *Dryopteris varia* (L.) Kuntze complex, was recently clarified using single-copy nuclear DNA markers (Hori et al. 2014, 2015, 2018a), and analyses on several other complexes are in progress. However, even using molecular markers, we often cannot find progenitors or sexual diploids in Japan. In some cases, these can be found in neighboring mainland countries, but in other cases progenitor taxa may be extinct. Hori et al. (2018c) discovered several sexual diploid individuals of *Dryopteris* in southern China, and was able to show that these functioned as diploid progenitors in the *D. erythrosora* (D. C. Eaton) Kuntze complex in Japan which had frustrated taxonomists for many years (Hori et al. 2016, 2018b). This case supported the hypothesis of Guo et al. (2003): “The ancestral sexual species of many Japanese apogamous species occur in China, suggesting that these species might have migrated from China”.

In contrast to these successful clarifications, we are still struggling with more challenging complexes. The species of *Cyrtomium* (Dryopteridaceae) in Japan serve as an example: 12 native species and subspecies are all triploid apomicts, except for two subspecies of *C. falcatum* (subsp. *australe* and subsp. *littorale*, sexual diploid) and *C. devexiscapulae* (Koidz.) Ching (sexual tetraploid) (Ebihara et al. 2017b; Matsumoto 2003). Despite careful exploration of sexually reproducing individuals by examining spore number per sporangium in herbarium specimens collected in China, a large part of them are apomictic, and sexual plants were found only rarely in geographically restricted ranges (Mitsuta 1986, 1988). The *Pteris fauriei* Hieron. complex is a similar situation, and includes several endemic ‘species’ to Japan which are diploid or triploid apomicts (Ebihara 2016). The “Diploids-First Approach” (Beck et al. 2010) is a useful principle for studies on fern reticulate complexes that prioritizes identification of diploid progenitors, but we should mind that diploids are not always present nearby, including possible extinction in complexes capable of apomixis.

## Species concepts

It is clear that the recent increase of recorded taxa in Japan is largely due to the transition from a morphological species concept to biological or evolutionary ones. In many cases, taxonomic revisions were published after analyses of species complexes. Although no final consensus has been reached, strict application of neither the biological species concept nor the evolutionary species seems to be preferred by recent studies. In particular, autotetraploids, which should be treated as an independent species from diploids sharing the same genome according to the biological species concept (as they are reproductively isolated), are more often treated as an infraspecific variant. Ebihara et al. (2005, 2009b) circumscribed a new allotetraploid species *Vandenboschia hokurikuensis* Ebihara including populations originating from different genotypes (at least two, based on maternally inherited plastid sequences). In practice, every allopolyploid with a different combination of diploid progenitor species requires a name. Among the 199 sexual polyploid (tetraploid or higher) taxa known in Japan, evidence for judging autopolyploidy vs. allopolyploidy are available in 34 (17.1%). Only four of them [*Asplenium boreale* (Ohwi ex Sa. Kurata) Nakaike (Fujiwara et al. 2017), *Asplenium pekinense* Hance (Lin and Viane 2013), *Pteris deltodon* Baker (Jaruwattanaphan et al. 2013), and *Lepisorus tosaensis* (Makino) H. Itô (Fujiwara et al. 2018)] are probably autopolyploid (autotetraploid), and the remaining 30 taxa are likely allopolyploid. Some of currently accepted infraspecific taxa defined to correspond to ploidy levels [e.g. diploid *Lycopodium clavatum* L. var. *nipponicum* Nakai vs. tetraploid var. *asiaticum* Ching (Ebihara, 2016; Takamiya 1989); tetraploid *Deparia petersenii* (Kunze) M. Kato var. *petersenii* vs. hexaploid var. *yakusimensis* (H. Itô) M. Kato (Shinohara et al. 2003)] might be classified into different species if future studies clarify differences in their genomic combinations.

We are often faced with a problem about taxonomic treatment for apomictic races. As they are asexually reproducing, the biological species concept is not applicable, and in fact several studies had no other choice than to defer corresponding taxonomic treatment (e.g. Chen et al. 2014; Nitta et al. 2011). Another issue relates to their nomenclatural categories: i.e., should we describe apomictic races of hybrid origin as nothotaxa? Recircumscription of species recently made by Hori et al. (2018a) for the *Dryopteris varia* complex is probably the most easily acceptable decision, i.e., provide a non-hybrid species name for every apomictic race with a different combination of sexually reproducing progenitors. This treatment resulted in a split of *Dryopteris hikonensis* (H. Itô) Nakaike into three apomictic species, namely *D. hikonensis* s.s. (= *D. protobissetiana* K. Hori et N. Murak. × *D. varia*), *D. erythrovaria* K. Hori et N. Murak. (= *D. caudipinna* Nakai × *D. protobissetiana* × *D. varia*) and *D. subhikonensis* K. Hori et N. Murak. (= *D. protobissetiana* × *D. saxifraga* × *D. varia*).

There is no standard taxonomic treatment for the case in which both sexual races and apomictic races coexist in a single species. The categorization into species, infraspecies, or nothotaxa largely depends on our observations and experiences, and it is not always possible to strictly separate them. While in many cases both reproductive modes are not distinguished taxonomically (e.g., *Pteris oshimensis* Hieron., *Phegopteris connectilis* (Michx.) Watt, *Dryopteris chinensis* (Baker) Koidz.), the apomicitc race of *Pteris terminalis* Wall. ex J. Agardh. is distinguished at varietal rank (var. *fauriei* (Christ) Ebihara et Nakato, Ebihara et al. 2017c) considering its morphological distinctness and the failure to find any unique alleles in the apomictic race. It should be noted that we treated some individuals originating from hybridization between an apomictic race and sexual species which have low spore germination rates and/or do not grow vigorously in the wild as nothotaxa in FernGreenList (Ebihara et al. 2017a).

## Gametophytes

Our knowledge about gametophytes of ferns and lycophytes is considerably limited compared with that of sporophytes; nevertheless, Japan is one of the world’s most information-rich areas for fern gametophytes. Two dennstaedtioid genera, *Coptodipteris* (‘Coptidipteris’) and *Fuziifilix* described by Nakai and Momose (1937) are some of the only genera to have been segregated based on gametophyte characters, even though they should probably be merged into one of the two clades of *Dennstaedtia* (Perrie et al. 2015). Momose (1967) observed and illustrated gametophytes of ca. 330 species mostly native to Japan using plants cultured from spores. Although his cultures were only successful almost exclusively for cordate gametophytes, subsequent researchers observed non-cordate gametophytes which grow and maturate typically slower than cordate ones [e.g., Hymenophyllaceae (Yoroi 1972, 1976, 1992), *Haplopteris* (Pteridaceae) (Yoroi 1975), *Pleurosoriopsis* (Polypodiaceae) (Ebihara et al. 2019c, Masuyama 1975), and *Botrychium* (Ophioglossaceae) (Takahashi and Imaichi 2007)].

Ferns and lycophytes are unique amongst vascular plants for having gametophytes and sporophytes that can grow separately from each other for long periods of time. Ebihara et al. (2010) established a country-wide DNA barcode library (plastid *rbcL* and *trnH*-*psbA* regions, covering ca. 94% of native fern and lycophyte taxa in Japan) which is quite useful for identification of gametophytes in the wild. Ebihara et al. (2013) demonstrated that non-cordate gametophytes frequently grow ‘independently’, or far from, their counterpart sporophytes, while cordate ones are almost always sympatric with conspecific sporophytes. This is also true for independent filamentous gametophytes of *Vandenboschia* as mentioned above. Furthermore, some of the independent gametophytes may completely lack sporophytes in Japan, including *Haplopteris* sp. from Yakushima Island (Kuo et al. 2017) and *Lomariopteris* sp. from Iriomote Island, which was first discovered by Ebihara et al. (2013), and still lacks a closely related sporophyte despite global taxonomic sampling (Kuo et al. 2018a; Kuo, personal communication). It is expected that expanded sampling of gametophytes in the wild will reveal more and more independent populations. Identification using DNA barcodes has also enabled detection of the fungal associations of wild fern gametophytes (Ogura-Tsujita et al. 2013, 2016, 2019). Although Ogura-Tsujita et al. (2013, 2016, 2019) discussed a possible correlation between gametophyte cushion structure and arbuscular mycorrhizal association, much wider taxonomic and geographical samplings are necessary for understanding such evolutionary trends as well as for comparison with symbiotic fungi in sporophytes (reviewed by Lehnert et al. 2016; Pressel et al. 2016). Finally, Dassler and Farrar (2001) posited several interesting hypotheses about advantages of non-cordate gametophytes in long-distance colonization and formation of island floras that could be effectively tested in the Japanese archipelago.

## Characters for taxonomic identification

Ebihara (2016, 2017) published matrices of major morphological characters frequently used for identification for all 721 native ferns and lycophytes in Japan. All quantitative characters used in Ebihara (2016, 2017) were newly measured by the members of the Nippon Fernist Club in ten randomly selected herbarium specimens per taxon for the present study. As these data are much more useful in an electronic form than as printed material, we provide the data file (ESM 3) formatted for Lucid 3.3 interactive key software, which has a free version available at http://www.lucidcentral.com/en-us/home.aspx.

## Future perspectives

Although none of the genera accepted in PPGI (Pteridophyte Phylogeny Group 2016) are endemic to Japan, recent studies have revealed several infrageneric clades with distribution centered in and/or having the highest diversity there. *Spicantopsis*, the most recently resurrected genus in Blechnaceae, is subendemic to Japan (2 endemic species and 1 species in Japan and Taiwan), and its ancestor is estimated to have migrated from North America to East Asia ca. 85 mya (Molino et al. 2019). Apart from *Spicantopsis*, we could find at least three groups exhibiting significant diversity in Japan: *Deparia* subsect. *Athyriopsis* (Kuo et al. 2016, 2018b), “Clades VI + VII” of *Lepisorus* (Wang et al. 2010), and the “the HYSUFI clade” of *Polystichum* (Le Péchon et al. 2016). Clarification of the speciation and biogeographic histories of such noteworthy groups is expected to contribute to understanding the formation of the Japanese flora in particular and floras of continental islands generally.

Regional floras are most useful not when used in isolation, but in comparison with those of neighboring regions in a global context. In widespread species or species complexes, we often encounter difficulty when applying the results of local studies with limited geographical coverage to the regional flora of unsampled areas. As Shrestha and Zhang (2015) demonstrated in Chinese populations, *Huperzia serrata* (Thunb.) Trevis. sensu lato (Lycopodiaceae) does not occur as a single species but rather an aggregation of multiple biological units, and this was supported by DNA content analyses using populations in Aichi Prefecture of Japan (Aman et al. 2011). Unfortunately, these findings were not reflected in FernGreenList ver. 1.0.1 (Ebihara et al. 2017a), which recognized only *Huperzia serrata* s.l., as we could not reliably synthesize these results for applying to Japanese populations. Likewise, there are a number of native species or groups in Japan which are awaiting careful comparison with published results mostly generated in China and other Asian countries [e.g. *Pteridium* (Zhou et al. 2014), *Hymenasplenium* (Xu et al. 2018), and *Leptochilus* (Zhang et al. 2019)].

In contrast to the consensus on familial classification of ferns and lycophytes nearly achieved in PPGI (Pteridophyte Phylogeny Group 2016), the generic classification proposed in the system requires a good deal of updating and emendation based on newly generated data. For example, the 30-genus system of Thelypteridaceae adopted by PPGI seems hardly applicable to the species in Japan as it would result in recognition of the nothogenus × *Chrinephrium* (× *C. insulare* (K. Iwats.) Nakaike), as well as uncertain placement for species formerly included in *Parathelypteris* (e.g., the material of “*Parathelypteris nipponica*” from China used in the phylogeny of He and Zhang (2012) and Almeida et al. (2016) is of dubious identification). It is important to publish data on East Asian material with accurate identification as the basis for generic recircumscription in the forthcoming “PPGII”.

Accumulation of data on ploidy and reproductive modes, along with recognition of proper taxonomic units and their distribution, has enabled us to discuss various characteristics of ferns and lycophytes such as the proportion of polyploids and the proportion of apomicts, and to compare these characteristics with those of other information rich areas. Combined analyses using the occurrence data provided by the present study and various kinds of geographic and environmental variables may yield further new insights, but here we confine our report to preliminary results.

We finally note an interesting finding, which is not evident in Fig. 1: a hotspot of diploid progenitors which are probable palaeoendemics at a riverside site in Amami-oshima Island, central Ryukyu. The hotspot is represented by at least four examples: (1) *Polystichum obae* Tagawa (Dryopteridaceae), an endemic diploid species (Takamiya 1996) with a very small population, is sister to a clade consisting of tetraploid *P. polyblepharon* (Roem. ex Kunze) C. Presl + diploid *P. parvipinnulum* Tagawa (Tsai and Shieh 1985) in a recent plastid phylogeny (Le Péchon et al. 2016); (2) *Coniogramme gracilis* Ogata (Pteridaceae), a diploid species (Kurita 1972) endemic to the island is presumably a progenitor of widespread tetraploid *C. japonica* (Thunb.) Diels (Kurita 1963); (3) *Diplazium amamianum* Tagawa (Athyriaceae), a diploid species (Takamiya et al. 1999) is endemic to the island, and sister to widespread apomictic species *D. hachijoense* Nakai in plastid phylogenies (Ebihara 2011; Wei et al. 2013); (4) *Pteris oshimensis* (Pteridaceae), a widespread species, has a sexual diploid found only in the Amami Islands in contrast to widespread apomictic races (Nakato and Ebihara 2016). The pattern—sexual diploids confined to the Amami Islands with widespread, closely related polyploids—shared by these four pairs strongly suggests that the island is a relic for ancestral diploids. However, this may be but one of many outcomes to come from analysis of the datasets made available here. It is highly expected that further analysis will result in robust evolutionary hypotheses accounting for the spatial distribution of diversity of the ferns and lycophytes of Japan, made possible by the accumulation and integration of large amounts of reliable information.
