# Correction to: An update and reassessment of fern and lycophyte diversity data in the Japanese Archipelago

**DOI:** 10.1007/s10265-019-01141-7

**Published:** 2019-10-18

**Authors:** Atsushi Ebihara, Joel H. Nitta

**Affiliations:** 1 Department of Botany, National Museum of Nature and Science, 4-1-1 Amakubo, Tsukuba, Ibaraki 305-0005 Japan; 2 Department of Botany, National Museum of Natural History, Smithsonian Institution, Washington, DC 20013 USA

## Correction to: Journal of Plant Research 10.1007/s10265-019-01137-3

In the original publication of this article, the affiliation of one of the authors was listed incorrectly as “Smithsonian Institute”. The correct affiliation is “Smithsonian Institution”.

